# Health Literacy in Pregnant Women: A Systematic Review

**DOI:** 10.3390/ijerph18073847

**Published:** 2021-04-06

**Authors:** Farah Nawabi, Franziska Krebs, Vera Vennedey, Arim Shukri, Laura Lorenz, Stephanie Stock

**Affiliations:** Institute of Health Economics and Clinical Epidemiology, University Hospital Cologne (AöR), 50935 Cologne, Germany; franziska.krebs@uk-koeln.de (F.K.); vera.vennedey@uk-koeln.de (V.V.); arim.shukri@uk-koeln.de (A.S.); laura.lorenz@uk-koeln.de (L.L.); stephanie.stock@uk-koeln.de (S.S.)

**Keywords:** health literacy, pregnancy, lifestyle, health behavior, systematic review

## Abstract

Health literacy plays a crucial role during pregnancy, as the mother’s health behavior influences both her own health and that of her child. To the authors’ best knowledge, no comprehensive overview on evidence of the health literacy of pregnant women and its impact on health outcomes during pregnancy exists. Therefore, this review aims to assess health literacy levels in pregnant women, whether health literacy is associated with outcomes during pregnancy and whether effective interventions exist to improve the health literacy of pregnant women. A systematic literature search was conducted in PubMed and EBSCO, resulting in 14 studies. The results show mixed levels of health literacy in pregnant women. Limited health literacy is associated with unhealthy behaviors during pregnancy. Mixed health literacy levels can be attributed to the recruitment site, the number of participants and the measurement tool used. Quality assessment reveals that the quality of the included studies is moderate to good. The review revealed that randomized controlled trials and interventions to improve health literacy in pregnant women are rare or do not exist. This is crucial in the light of the mixed health literacy levels found among pregnant women. Healthcare providers play a key role in this context, as pregnant women with limited health literacy rely on them as sources of health information.

## 1. Introduction

Health literacy is widely defined as “[…] people’s knowledge, motivation and competences to access, understand, appraise, and apply health information in order to make judgments and take decisions in everyday life concerning healthcare, disease prevention and health promotion to maintain or improve quality of life during the life course” [1] (p. 3). Despite the availability and accessibility of health information, considerable parts of the population still engage in risky health behavior such as insufficient physical activity, unbalanced nutrition and smoking. These risk factors are associated with chronic diseases such as diabetes, which cause more than 75% of deaths worldwide [2]. Limited health literacy is an important driver in health disparity as it is associated with insufficient self-management and worse health outcomes in chronic diseases [3]. Individuals with limited health literacy have more emergency department visits, more and longer hospital stays, worse outcomes in healthcare, and lower utilization of preventive services than people who show an adequate level of health literacy [4]. A multinational study conducted in Europe, for example, revealed that 12% of the respondents possessed inadequate health literacy and 47% displayed limited health literacy, with major differences across countries [5].

Adequate access, understanding and application of health information is important, especially with regard to high-risk health behaviors and in vulnerable situations. One example of such a situation where health behavior becomes particularly important is pregnancy, since in this phase behaviors affect the health of both the woman and the fetus. During pregnancy, women are confronted with a variety of health information from different sources [6]. This information entails recommendations regarding health behavior. Despite the existence of evidence-based recommendations and information materials, pregnant women with limited health literacy are less likely to take folic acids during pregnancy or engage in prenatal care at a later gestational age, and have more hospital stays [7]. Moreover, these women are less likely to engage in breastfeeding for the first two months after birth [8]. At the same time, women with adequate health literacy levels have a better understanding of the dangers of smoking during pregnancy [9]. For women with limited health literacy, written information on antenatal services is more difficult to understand. As such, these women are less likely to make informed medical decisions [10]. Since maternal lifestyle during pregnancy influences child health in later years through epigenetic programming, it is essential to develop approaches to improve health literacy among pregnant women in order to keep both mother and child healthy.

In recent years, research has mainly focused on the assessment of health literacy levels among the general population and particular at-risk groups such as older people, immigrants and people with a low socio-economic status, or has only taken particular focus on gestational weight gain [11] and reproductive health [10]. Despite the growing recognition of the importance of health literacy, there has not yet been any comprehensive literature review on the association between health literacy levels among pregnant women and health outcomes during pregnancy. Additionally, it is unclear whether effective interventions exist that improve health literacy among pregnant women.

Therefore, a systematic review was conducted to assess (1) health literacy levels in pregnant women; (2) whether health literacy is associated with outcomes during pregnancy; (3) whether interventions exist to improve the health literacy of pregnant women.

## 2. Materials and Methods

### 2.1. Data Sources

A bibliographic search was conducted in PubMed and EBSCO. In addition to this, a hand search was conducted using Google Scholar. The search terms were kept general in order to maximize search sensitivity. Table 1 displays the search strategies, as well as the inclusion and exclusion criteria. We included studies published in the last ten years (2009–2019, with an updated search in 2020), as we wanted to obtain recent literature and health literacy has become an increasingly relevant field of research in the last decade. Studies had to be in English and had to measure health literacy among pregnant women using at least one validated quantitative tool. We only included studies that measured health literacy as a multidimensional concept, and excluded studies that exclusively assessed knowledge. The inclusion and exclusion criteria can be found in Table 1.

Two authors (F.N., F.K.) independently screened and evaluated all the abstracts. Where applicable, the articles were subsequently included for full-text review and data extraction.

### 2.2. Data Extraction

Relevant information from the retrieved studies, including the general characteristics of the study and the quantitative results, was extracted based on a predefined data extraction tool. Two researchers (F.N., F.K.) independently extracted information related to the authors and the country of origin, the year of publication, the data collection setting, and factors that might have an impact on the health literacy level, such as the recruitment strategies, the underlying definition of health literacy, and the health literacy tool used.

Quantitative results were extracted as provided in the studies, e.g., as percentages of women with limited or adequate health literacy, average health literacy scores, results of tests for group differences, and the respective significance levels. Quantitative data were extracted independently by two reviewers (F.N., A.S.). Due to the diversity of the studies’ characteristics and the way the results were presented, the data were not summarized quantitatively in a meta-analysis.

### 2.3. Quality Assessment

The methodological quality of all the studies included in the review was assessed using standardized checklists. Since this review included different types of study design, a number of different quality assessment tools were used. For randomized controlled trials (RCT), we used the RoB 2.0 risk assessment tool provided by the Cochrane group [12]. This tool covers five domains of bias, focusing on trial design, conducting and reporting. Each domain entails three to seven aspects, for which the risk of bias is rated as ‘Low’, ‘High’ or ‘Some concerns’. A study is rated as having an overall high risk of bias if any of these aspects is rated as having a ‘High risk’ of bias. For cross-sectional studies, we applied the Appraisal tool for Cross-Sectional Studies (AXIS) [13]. This tool has a set of 20 questions that cover every section of a cross-sectional study, from the introduction to discussions. Each question is answered using ‘Yes/No’ or ‘Don’t know/Comment’. The AXIS does not provide an overall assessment of a study. Two reviewers (F.N, F.K) rated the study quality independently, and any conflicts were resolved through discussion.

## 3. Results

### 3.1. Study Selection

**Figure 1:**
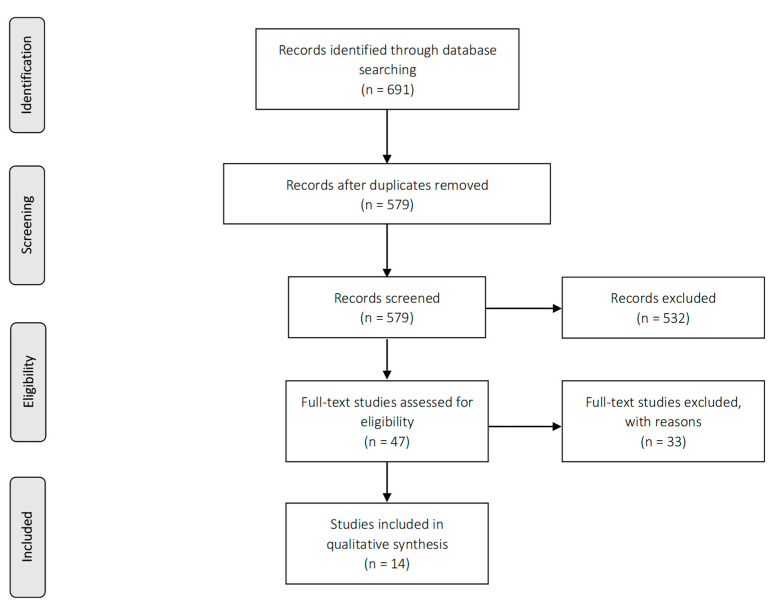
PRISMA flow chart.

Figure 1 shows the flow chart used for study selection. 691 studies were identified in total. 112 duplicates were removed. The titles and abstracts of the 579 remaining studies were then screened. 532 of these studies did not match the inclusion criteria, which left 47 studies for full-text screening. Eventually, 14 remained to be included in this review after an updated search in August 2020. No additional studies were retrieved through hand search.

### 3.2. Study Characteristics

The included studies are summarized in Table 2. Thirteen of the 14 studies were cross-sectional in nature. One study used an experimental design, comparing a treatment group receiving an interactive patient education tool for prenatal screening and diagnosis to a control group that was receiving standard care counselling [14].

Most of the studies were conducted in Europe, Canada and the USA. The sample size of study participants ranged from *n* = 34 to *n* = 4999. Except for a minimum age of 18 years, the inclusion criteria for the study participants varied across the studies. The time of gestation at inclusion varied, with some studies only including women at the beginning of the pregnancy [15] and others including women at the end of pregnancy [21]. The studies did not include or exclude women based on their ethnicity or educational attainment. Further details on these characteristics are provided in Supplementary Table S1. Most of the studies required the women involved to be healthy [15,17]; however, some also included women at risk of a condition [31,32], depending on the main outcome of the study. Primary outcomes also varied across the studies.

Three of the studies used the REALM, four studies used the S-TOFHLA, three the NVS and five the BHLS. Some studies utilized two instruments. One study used the 25-item version of the HLS-EU (Table 3). Since each of these tools uses different terms to define health literacy scores, this paper summarizes the definitions as ‘Limited’ (original: Limited, Inadequate, Insufficient, Low), ‘Marginal’ (original: Marginal, Medium), and ‘Adequate’ (original: Adequate, Sufficient, High).

#### 3.2.1. Objective One: Health Literacy Levels in Pregnant Women

The studies included in this review (Table 4) show mixed findings regarding health literacy levels among pregnant women. Two studies report that health literacy levels among pregnant women are limited based on the REALM [14,33], which corresponds to 4th–6th grade reading level (Table 2). By contrast, about 85% of the participants in the study conducted by Duggan et al. in 2014 demonstrated adequate levels of health literacy using the REALM [19].

Based on the utilization of the S-TOFHLA, the participants in three of the studies scored adequately [27,29,35]. Similarly, Delanoe et al. [17] found that health literacy levels in their population were adequate using both the S-TOFHLA as an objective tool and the BLHS as a subjective tool (Table 4). The study by You et al. also reveal adequate health literacy levels. However, the scoring in their study reach up to 100, indicating that this study is likely to have used the TOFHLA and not the short version of it as stated in their study [35].

Lupattelli et al. [21] conducted a transnational study. The overall health literacy levels using BHLS were mixed: 54.5% scored high, 40.3% scored marginal and 5.2% scored low. Both studies from van Schendel et al. [31,32] depict adequate health literacy in pregnant women using the BHLS. A further study from Delanoe et al. [15] demonstrated mixed results using both the BHLS (marginal health literacy) and NVS (adequate health literacy).

Sheinis et al. [26] split the health literacy results of their study population into two age groups, both of which revealed adequate health literacy.

#### 3.2.2. Objective Two: Effect of Health Literacy on Outcomes during Pregnancy

Health literacy is associated with a variety of outcomes, which can be categorized into ‘Beliefs/attitudes’, ‘Knowledge’ and ‘Lifestyle’ (Table 5).

Limited health literacy is associated with more negative beliefs regarding medicine [19] and a higher level of residual anxiety when receiving normal results for genetic tests [32]. This is due to the fact that women did not fully understand the normal test results they were given, which indicated that the fetus was less likely to suffer from a form of trisomy [32]. In contrast, adequate health literacy was associated with making an informed choice with regard to prenatal testing. In turn, informed choices were associated with lower levels of decisional conflict and anxiety [31]. Women with limited health literacy believed that the health provider was responsible for their infants’ health [29] and made more use of interpersonal information sources such as information provided by health professionals, friends and family [27]. Delanoe et al. [17] concluded that health literacy does not influence the intention to use a decision aid for trisomy 21 screening. All pregnant women are influenced to the same degree by socio-cognitive factors when it comes to using a decision aid for screening. A different study by Delanoe et al. [15] showed that only subjective health literacy was associated with the intention to use a decision aid for prenatal screening. However, this result does not apply when considering objective health literacy. The NVS was not discriminative enough, leading to the conclusion that the women’s own perception of health literacy influences their intention to use a decision aid.

Smoking behavior was addressed in one study, which found that women with limited health literacy smoke during pregnancy [21]. Moreover, limited health literacy was associated with higher risk perception and negative beliefs with regard to medication, and non-adherence to prescribed medicines [21]. One study concluded that health literacy is significantly and positively associated with a health promoting lifestyle (spiritual growth and interpersonal relations) and negatively associated with the intake of antidepressants and flu vaccines. Moreover, women with planned pregnancy and who used medication during their pregnancy have a high level of health literacy [23].

Women with limited health literacy gave more wrong answers in a questionnaire on the risks, benefits and safety of Tuberculosis and Hepatitis B vaccines [33], and an adequate health literacy level was associated with better scores in a preeclampsia questionnaire [35]. However, the latter association was not significant in the multivariable regression, which can be explained by the small number of participants who had limited health literacy [35]. Higher health literacy scores correlated positively and significantly with knowledge of age-related pregnancy risks in the study by Sheinis et al. [26]. However, in a different study by Sheinis et al. health literacy was not associated with knowledge of trisomy 21 [24].

#### 3.2.3. Objective Three: Interventions to Improve Health Literacy among Pregnant Women

None of the studies included in the review were aimed at improving health literacy among pregnant women. One study conducted an RCT aimed at improving knowledge of prenatal genetic testing among pregnant women [14]. The women in the intervention group received an interactive educational tool, while the control group received standard care. The results showed that, regardless of health literacy levels, women in both groups had a similar improvement in knowledge scores (Table 5). This indicates that the intervention did not particularly improve health literacy, but was still health-literacy-sensitive.

#### 3.2.4. Quality Assessment

All the studies included in this review met at least 13 out of the 20 possible AXIS points (range: 13–19). Two of the studies achieved 13 points, three achieved 14, one achieved 15, one achieved 16, five achieved 17, one achieved 18 and another achieved 19 points.

All the studies fulfilled the quality criteria reflected in the inclusion criteria of this review, such as specifying the target group and using a validated measurement tool. All the studies reported the use of a precision estimate (e.g., *p*-values), either directly in the Methods sections or indirectly in the results presented in the study. Additionally, all of the studies included in the review provided a discussion of their own limitations. However, some of the studies did not meet items of the quality assessment tool that have a significant impact on how a study is conducted. Eight of the studies included in the review did not provide grounds for their sample sizes. Only one study addressed and categorized non-responders. The response rate raised concerns with regard to non-response bias in five of the studies. Most studies (*n* = 8) applied convenience sampling. Twelve studies provided indications that there might be a lack in the representativeness of the sample.

The overall quality of the included RCT was rated as ‘High risk’, since the points “Risk of bias in measurement of the outcome” and “Risk of bias due to deviations from the intended interventions” were rated as having high risk of bias. An extended overview of the quality assessment can be found in Supplementary S2.

## 4. Discussion

To the best of our knowledge, this review is the first to review systematically overall health literacy among pregnant women. We identified 14 studies on the health literacy of pregnant women, measured quantitatively with at least one validated tool. These studies also report on the effect of health literacy on beliefs/attitudes, knowledge and lifestyle during pregnancy.

Regarding the first objective of this review, the studies show mixed results regarding the health literacy levels of pregnant women. The majority of the studies included in the review indicate that the women surveyed have an adequate health literacy level. However, the women in the studies included in this review were recruited mainly from western high-income countries and cities, or web-based panels to which they signed up willingly. This may lead to the assumption that these groups have adequate health literacy than the general population [17]. In contrast, research suggests that women in countries below poverty level are more likely to possess only limited health literacy [33].

Nevertheless, some studies display limited health literacy levels in the target group. This can be attributed to the use of different measurement tools. Even though all the tools used have been validated for measuring health literacy, they measure the concept differently: while the BHLS and HLS-EU measure health literacy subjectively, the NVS, S-TOFHLA and REALM are objective measures. Health literacy research indicates that when both objective and subjective tools are used to measure health literacy within the same population, conflicting results can occur, since associations with other variables emerge differently when using objective tools to when using subjective ones [15,41,42,43,44]. Even within the objective measures, the tools use differing methods to assess health literacy. While the NVS measures numeracy and reading skills based on a nutrition label from an ice cream container, the S-TOFHLA also measures these skills using a fill-in-the-blanks text with a choice of words and the REALM measures health literacy by means of an oral reading and recognition test. Moreover, studies repeatedly point out that tools might not have been sensitive or discriminative enough [15,29]. Additionally, the majority of the studies included in the review did not use the cut-off points to display different health literacy levels, as suggested in the manuals of the original tools. The studies mostly condensed the ‘High’, ‘Medium’ and ‘Low’ cut-offs for health literacy to just ‘High’ and ‘Low’. Other studies [15,17,26,29] offered an overall mean score, which impedes comparisons across studies.

The definition and level of education in the samples of the included studies is heterogeneous. Therefore, specific sub-group-analysis based on education were not feasible. Studies depict that the majority of participants had some form of higher education, such as college or university degree. Still, health literacy levels are not consistently adequate throughout the studies. High educational attainment alone does not translate to adequate health literacy levels [21] and is not sufficient to prepare pregnant women for events that occur during pregnancy, such as counselling for prenatal genetic testing [14]. Hence, health literacy sensitive interventions during pregnancy could be beneficial for all pregnant women, regardless of their educational and health literacy levels [14].

The studies included in this review depict associations between health literacy and outcomes within the categories of health beliefs and attitudes, knowledge and lifestyle (objective two). Women with limited health literacy had more negative beliefs regarding medication [19], whereas women with adequate health literacy made more informed choices with regard to prenatal testing [31]. Women with adequate health literacy scored better in knowledge-based questionnaires. The positive association between adequate health literacy and adequate knowledge is supported by other studies [45]. Concerning lifestyle, one study found a positive association between limited health literacy and probability of smoking. Health literacy research confirms this association, as well as other negative behaviors that go hand-in-hand with limited health literacy [46].

Although research in the field of health literacy has gained more attention in recent years, it was not possible to identify a study that was aimed at improving health literacy among pregnant women, and that therefore addressed our third research objective. Only one study conducted an RCT with an intervention in order to improve knowledge that resulted in health literacy sensitivity, meaning women benefitted equally regardless of their health literacy levels [14]. This is striking, since the majority of the studies stress the importance of health-literacy-sensitive actions in improving health literacy among pregnant women. RCTs on health literacy actions should also consider facilitators and barriers for implementation such as the time required for clinicians to provide adequate consultation, improvement of health information regarding health literacy and the format of material provided (e.g., written or web-based). Enabling people to find, understand, appraise and apply health information is also highly relevant to ensure the provision of truly informed consent.

The studies included in the review reveal that the role of health professionals during pregnancy is crucial, since they provide women with prenatal counselling. It is therefore crucial for healthcare providers to ensure that women understand the health information they are given. Women with limited health literacy might benefit from additional explanation for genetic testing both prior to the test and after receiving normal test results [32], as women with an adequate level of health literacy are more likely to make an informed choice with regard to whether or not to have NIPT [31]. Medicine adherence is also dependent on the healthcare providers’ responsiveness to the women’s ability to understand health information [21]. This is particularly the case for women with limited health literacy, who mainly rely on the information provided by healthcare providers because they do not use the Internet to find health information. Instead, they are more likely to rely on interpersonal communication and, primarily, on their healthcare providers, because they lack the skills required to find and understand health information from other sources [29]. This reliance is also likely to result in a ‘powerful others’-oriented fetal health locus of control, meaning that women with limited health literacy believe their healthcare provider is responsible for the infants’ health [29]. Research already suggests that interventions are needed to improve health literacy in patients from a systems perspective, meaning that health professionals need to improve their communication skills towards being more health-literacy sensitive [47,48]. Visscher et al. identified three factors that increase the likelihood of health literacy interventions being effective: (1) the interventions’ activities are tailored to the particular needs of people with limited health literacy, (2) they target interactive and/or critical health literacy skills (as opposed to being purely knowledge-based) and (3) they present information in an understandable way [49].

### Limitations

The results of this review must be viewed in the light of several limitations. Firstly, the studies included in the review were mainly of moderate quality. This is critical to the validity of this review, as studies with a good level of evidence are lacking. This can be attributed to the nature of the study designs, namely cross-sectional studies. However, the majority of the studies that exist in the field have a cross-sectional design, which indicates the need for RCTs in the field of health literacy among pregnant women. This way, causal associations can be evaluated. Additionally, the majority of studies indicate that the sample might not be representative, which is attributable to the sample size or sampling method. Moreover, educational level was not categorized in a standardized manner, which hindered separate analyses based on this characteristic. Secondly, no interventions exist for improving health literacy among pregnant women. Due to the lack of such studies, it was not possible for us to achieve the third objective of this review. Thirdly, we did not include databases that cover midwifery in our search. This might have led to the omission of relevant literature. Fourthly and finally, the decision to limit eligible literature to that published in English might also have led to the neglect of important studies.

## 5. Conclusions

The results of this review indicate that health literacy levels in pregnant women vary across different studies. Even though most studies were conducted in western countries, limited health literacy was present and might be due to the socio-economic status of the study participants. Some of the studies included in the review recruited women from clinics that predominantly catered to low-income patients, which might be attributable to the low socio-economic status of such women. However, data formats did not allow for analyses, e.g., based on educational level. The association between health literacy and different health outcomes that are present in the studies of this review are well known for other populations as well. Health literacy research suggests that inadequate health literacy is associated with smoking, higher risk perception and negative beliefs about medication and non-adherence to prescribed medicines, which is also true for pregnant women. With the studies depicting low levels of health literacy, it is striking that no interventions exist to improve health literacy during pregnancy, not only because an adequate level of health literacy is important for the health of the women involved, but also because health literacy levels influence other health outcomes and behaviors during pregnancy, which will most likely affect the unborn child’s health and development. Additionally, to ensure informed consent in medical decision-making conforms to legal and ethical requirements, the effects of health literacy on providing informed consent should be investigated. Overall, randomized-controlled intervention studies are needed to build evidence-based strategies to increase health literacy for better health among pregnant women.

## Figures and Tables

**Table 1:** Inclusion and exclusion criteria and search strategies.

Inclusion criteria	- Pregnant women at any week of gestation - English literature - Quantitative studies - All study designs - Health literacy as an outcome - General/overall health literacy - Health literacy measure with at least one validated tool - Assessment of one of the following: Health literacy levels among pregnant women The effects of health literacy on outcomes during pregnancy Interventions that (in)directly affect (improve) health literacy
Exclusion criteria	- Preconception - Postnatal, after birth - Reproductive health - Languages other than English/German - PhD theses - Qualitative studies - Topic-specific health literacy
PubMed	(health literacy) AND pregnan * Sort by: Best Match Filters: published in the last 10 years (2009–2019 with updated search in 2020)
EBSCO	health literacy AND pregnan * Limiters—Publication Year: 2009–2019 (with updated search in 2020)

*= truncated search term.

**Table 2:** Study characteristics.

* 1st Author *	Year	Country under Study	Study Design	Eligibility Criteria	Health Literacy (HL) Definition	Measure	*n* in Analysis	Sampling & Recruitment
*Delanoe* [15]	2016	Canada	Cross-sectional, embedded in a questionnaire pilot test	≥18 years old; second trimester of pregnancy; no high-risk pregnancy (excluding down syndrome risk)	Nutbeam (2000) [16]	NVS; BHLS	45	Convenience sample from three clinical sites
*Delanoe* [17]	2016a	Canada	Cross-sectional	≥18 years old; ≥16 weeks pregnant; no high-risk pregnancy; decided about prenatal screening	Nutbeam (2008) [18]	S-TOFHLA; BHLS	346	Web-based survey
*Duggan* [19]	2014	Ireland	Cross-sectional	≥18 years old; English-speaking; no visual or aural impairments	Ad Hoc Committee on Health Literacy for the Council on Scientific Affairs (1999) [20]	REALM	404	Convenience sample from a university hospital
*Lupattelli* [21]	2014	Australia, Austria, Canada, Croatia, Finland, France, Iceland, Italy, Netherlands, Norway, Poland, Russia, Serbia, Slovenia, Sweden, Switzerland, UK, USA, some South American countries	Cross-sectional	Any week of gestation	Nielsen-Bohlman, Panzer, Kindig (2004) [22]	BHLS	4999	Web-based survey Advertisement was placed on websites used frequently by pregnant women, inviting them to take part in the survey
*Sahin* [23]	2020	Turkey	Cross-sectional	≥18 years old; Turkish-speaking	Definition provided without source	HLS-EU-25	326	At a hospital
*Sheinis* [24]	2018	Canada	Cross-sectional	Low and high-risk obstetrics patients; English-speaking	Safeer and Keenan (2005) [25]	NVS	139	Convenience sample from a hospital
*Sheinis* [26]	2018a	Canada	Cross-sectional	Primipara; receiving prenatal care hospital of conduct and attending prenatal visit in a low risk obstetrics clinic; English-speaking	None provided	NVS	218	Convenience sample from a hospital
*Shieh* [27]	2009	USA	Cross-sectional	≥18 years old; English-speaking; publicly funded or no health insurance	Kutner, Greenberg, Jin, Paulsen (2006) [28]	S-TOFHLA	143	Convenience sample from a prenatal clinic in an urban community that predominately catered to low-income patients
*Shieh* [29]	2010	USA	Cross-sectional	≥18 years old; English-speaking; government subsidized health insurance or no health insurance	Rootman (2004) [30]	S-TOFHLA	143	Convenience sample from a prenatal clinic in an urban community that catered to low-income patients
*Van Schendel* [31]	2016	Netherlands	Cross-sectional, survey of HL embedded in pre/post design	≥18 years old; increased risk of trisomy; >10 weeks pregnant; no multiple pregnancies, no vanishingtwin, no structural fetal anomalies, no maternal history of malignancy or chromosomal abnormality	None provided	BHLS	1091	Eight prenatal diagnosis centers
*Van Schendel* [32]	2017	Netherlands	Cross-sectional	See van Schendel, 2016	None provided	BHLS	682	See van Schendel, 2016
*Wilson* [33]	2012	Jamaica	Cross-sectional	≥18 years old; attending the clinic for prenatal care	Baker (2006) [34]	REALM	34	Convenience sample from two community health centers that predominately catered to low-income patients
*Yee* [14]	2014	USA	Ranomized Control Trials (RCT)	≥18 years old; 6th–26th weeks pregnant; not undergone any prenatal testing; English-speaking; no multiple gestations	None provided	REALM	150 (75/75)	During routine prenatal visits in a clinic
*You* [35]	2012	USA	Cross-sectional	≥18 years old; 18th–40th weeks pregnant; English-speaking; no visual or aural impairments	None provided	S-TOFHLA	110	Convenience sample from a university clinic

NVS = Newest Vital Sign; BHLS = Brief Health Literacy Screener; S-TOFHLA = Short Test of Functional Health Literacy in Adults; REALM = Rapid Estimate of Adult Literacy in Medicine; HLS-EU-25 = Health Literacy Survey Europe Questionnaire.

**Table 3:** Tools used in the studies.

**Tool**	**Description**	**Scoring**
**REALM** [36]	This objective tool is an oral reading and recognition test with 66 medical terms. Every correctly pronounced word equals one point.	Total score: 66 0–44 is limited health literacy (6th grade or below); 45–60 is marginal health literacy (7th–8th grade); 61–66 is adequate health literacy (above 9th grade)
**S-TOFHLA** [37]	This objective tool measures both reading comprehension and numeracy. The reading part entails a fill-in-the-blank text that offers a choice of four words. The numeracy part uses hospital forms and labelled vials, and requires interpretation of such numbers.	Total score: 36 0–16 is limited health literacy; 17–22 is marginal health literacy; 23–36 is adequate health literacy
**NVS** [38]	This objective tool is based on an ice cream label. Patients have to answer a total of six questions related to the label: four requiring numeracy skills and two requiring reading skills.	Total score: 6 0–1 is the high likelihood of limited health literacy; 2–3 is the possibility of limited health literacy; 4–6 is adequate health literacy
**BHLS** [39]	This subjective screener consists of three questions concerning medical forms and information.	Total points: 12 0–5 is limited health literacy; 6–9 is marginal health literacy; 10–12 is adequate health literacy
**HLS-EU-25** [40]	This subjective tool covers the process of accessing, understanding, appraising and applying health-related information within the fields of healthcare, disease prevention and health promotion.	Total score: 125, without qualitative categorization of HL

**Table 4:** Studies that described health literacy levels in pregnant women.

1st Author	Tool	Result/Health Literacy Level	Remarks
Yee, 2014 [14]	REALM	43.3% with limited health literacy, 56.7% with adequate health literacy	One cut-off point, it is not apparent at which score
Duggan, 2014 [19]	REALM	15.3% with limited health literacy, 84.7% with adequate health literacy	One cut-off point at a score of >60 = adequate health literacy
Wilson, 2012 [33]	REALM	85% with limited health literacy, 15% with adequate health literacy	Study offers differentiated scores, which were taken together for comparability *
Shieh, 2009 [27]	S-TOFHLA	14.7% with limited health literacy, 85.3% with adequate health literacy	Cut-offs (>30 adequate health literacy) different to those suggested by the original tool
You, 2012 [35]	S-TOFHLA	9% with limited health literacy, 91% with adequate health literacy	Cut-offs (≥66 = adequate health literacy) different to those suggested by the original tool. It appears that the study uses the TOFHLA rather than S-TOFHLA, since scores go up to 100 instead of 36
Shieh, 2010 [29]	S-TOFHLA	Mean: 32.35 (5.14)	S-TOFHLA presented as mean score instead of health literacy distribution
Delanoe, 2016a [17]	S-TOFHLA	Median: 36	No further analysis with S-TOFHLA due to lack of variability. Cut-offs for BHLS different to those suggested by the original tool (>10 = adequate health literacy); no health literacy distribution for either tool
	BHLS	Median: 10	
Lupattelli, 2014 [21]	BHLS	45.5% with limited health literacy, 54.5% with adequate health literacy	Study offers differentiated scores, which were taken together for comparability *
Van Schendel, 2017 [32]	BHLS	6.8% with limited health literacy, 93.2% with adequate health literacy	One cut-off point, it is not apparent at which score
Van Schendel, 2016 [31]	BHLS	8.5% with limited health literacy, 91.5% with adequate health literacy	One cut-off point, it is not apparent at which score
Delanoe, 2016 [15]	BHLS	Median: 8/mean: 8.2 (1.6)	BHLS and NVS are each presented as one score instead of health literacy distribution
	NVS	Mean: 5.3 (1.6)/median: 6	
Sheinis, 2018a [26]	NVS	Mean: 4.5 (1.53) < 35 years old; Mean: 4.7 (1.39) ≥ 35 years old	NVS presented as means and cut-off was set at age (35 years)

* Note: For purposes of comparability, attempts were made to make the results of each study consistent. However, this was not possible because some studies (a) used different cut-off points than those suggested in the original tool or (b) used different statistical methods, and the original data were not available.

**Table 5:** Studies that indicated an association between health literacy and other outcomes during pregnancy.

Study	Outcome	Univariate Analysis		*p*-Value	Multivariate Analysis	*p*-Value
	**Beliefs/attitudes**					
Duggan, 2014 [19]	Women with limited HL have more negative beliefs regarding medicines, even when controlling for age and education. Note: Rather than being shown as a single score, negative beliefs aresplit into general harm and general overuse based on the Beliefs About Medicine questionnaire.	Comparison of means (t-test)			Multiple linear regression	
		General harm			DV: General harm	
		Limited HL: M = 11.85 (SD = 2.81)		<0.001	IV: Limited HL with	
		Adequate HL: M = 9.75 (SD = 2.11)			β = 1.73; 95% CI [1.11–2.34]	<0.001
		General overuse			DV: General overuse	
		Limited HL: M = 12.48 (SD = 2.63)		0.01	IV: Limited HL with	
		Adequate HL: M = 11.51 (SD = 2.73)			β = 0.95; 95% CI [0.19–1.70]	0.01
Van Schendel, 2017 [32]	Women with limited HL experience greater residual anxiety (using the State-Trait Anxiety Inventory (STAI) and Pregnancy Related Anxiety Questionnaire-Revised (PRAQ-R)) after receiving normal Non-Invasive Prenatal Testing (NIPT) results.				ANCOVA for women with normal NIPT results (covariate: STAI and PRAQ-R)	
					DV: Post-test-result STAI score	
					IV: HL	
					Limited HL: M = 31.6	0.047
					Adequate HL: M = 28.6	
					DV: Post-test-result PRAQ-R score	
					IV: HL	
					Limited HL: Data not shown	<0.001
					Adequate HL: Data not shown	
Shieh, 2010 [29]	Limited HL was inversely correlated with the ‘Powerful others’ dimension from the Fetal Health Locus of Control (FHLOC) scale, indicating that women perceive healthcare provider as the party responsible for the child’s health. No association was found between HL and the seeking of health information.	Correlation between HL and FHLOC: r = −0.28		0.003		
		Univariate linear regression				
		DV: Seeking of health information				
		IV: HL with β = −0.05		0.58		
Shieh, 2009 [27]	Pregnant women with limited HL used the Internet less frequently as a source of information. Women with limited HL tend to use interpersonal information such as healthcare providers and friends/family sources more frequently.	Fisher’s exact test				
		Frequent Internet use				
		Limited HL: 14.3%		0.007		
		Adequate HL: 46.7%				
Delanoe, 2016 [15]	Subjective HL, using the BHLS, was positively association with the intention to use a decision aid for prenatal screening (IDAPS). Objective HL was not significantly correlated with this.	Correlation between subjective HL and IDAPS: Rho = 0.32		0.04		
Delanoe, 2016a [17]	HL does not influence the intention to use a decision aid for trisomy 21 screening.	Bivariate ordinal logistic regression			Ordinal logistic regression	
					DV: intention	
		DV: intention level			IV: attitude, subjective norm, perceived control	
					(model I)	
		IV: STOFHLA		0.27	Adding moral, descriptive norm and anticipated regret leads to model II. Model I vs. model II:	
					Δ deviance = 41.33	<0.001
		IV: BHLS		0.52	Adding the BHLS to modell II leads to model III and:	
					Δ deviance = 0.63	0.43
Van Schendel, 2016 [31]	Women with adequate HL were more likely to make an informed choice concerning prenatal testing.	Univariate logistic regression			Multiple logistic regression	
		DV: Informed choice			DV: Informed choice	
		Covariate: Adequate HL with			IV: Adequate HL with	
		OR = 3.14, 95% CI [1.77–5.57]		<0.001	OR = 2.60, 95% CI [1.36–4.95]	0.004
	**Knowledge**					
Sheinis, 2018a [26]	HL correlated positively and significantly with knowledge of age-related pregnancy risks.	Correlation between HL and knowledge of age-related risks:			Multiple linear regression	
					DV: Knowledge score	
		r = 0.146		0.03	IV: HL with β = 0.261	0.027
Wilson [33]	Incorrect responses regarding the benefits and risks of the vaccines were more common among women with lower REALM scores.	By category of response (F-test)	REALM Score			
		Tuberculosi vaccine benefits				
		Correct	42.7			
		Partially correct	41.6			
		Incorrect	31.4	0.41		
		Tuberculosis vaccine risks				
		Correct	46.2			
		Partially correct	42.6			
		Incorrect	20.5	0.01		
		Hepatitis B vaccine benefits				
		Correct	45.6			
		Partially correct	42.5			
		Incorrect	30.6	0.13		
		Hepatitis B vaccine risks				
		Correct	45.5			
		Partially correct	44.3			
		Incorrect	21.9	0.01		
You, 2012 [35]	Women with adequate HL returned significantly better scores in a preeclampsia questionnaire. However, this association was not significant in the multivariate analysis.	Comparison of means (t-test)				
		Preeclampsia questionnaire score				
		Adequate HL: M = 44.6%		0.035		
		Marginal/inadequate HL: M = 29.6%				
Yee, 2014 [14]	Regardless of HL levels, women in both the education tool group and the standard care group demonstrated a similar improvement in knowledge scores.				Two-way ANOVA	
					Test scores (% correct)	
					Standard care	
					Limited HL: 39.7 (SD = 13.7)	0.81
					Adequate HL: 49.9 (SD = 15.0)	(Inter-action)
					Educational tool	
					Limited HL: 64.7 (SD = 13.7)	
					Adequate HL: 73.8 (SD = 13.3)	
Sheinis, 2018 [24]	HL was not shown to be a predictor of knowledge of prenatal screening for trisomy 21.				Multiple linear regression	
					DV: Knowledge of trisomy 21	
					IV: HL with β = 0.46	0.52
	**Lifestyle**					
Lupattelli, 2014 [21]	(1) Women with inadequate HL tend to smoke during pregnancy. (2) Women with inadequate HL have higher risk perception and negative beliefs regarding medication. (3) Non-adherence to prescribed medicines differed across HL groups.	(1) No smoking (%) Limited HL: 81.9, Marginal HL: 89.8, Adequate: 92.1 (2) Correlation between HL and belief sum score: Rho = −0.160 (3) Non-adherence (%) Limited HL: 25.0, Marginal HL: 22.5, Adequate: 19.2		(1) <0.05 (2) <0.01 (3) <0.001	Generalized estimating equations DV: Non-adherence IV: Limited HL with OR = 1.43, 95% CI [1.09–1.88] Covariates: region of residency, maternal age, educational level, employment status, immigrant status	
Sahin, 2020 [23]	There is a significant positive association between HL and aspects of health promoting lifestyle, and with a significant negative association between HL and intake of antidepressants and flu vaccines. Women with planned pregnancy and who used medication during their pregnancy have a high level of HL	Correlation between HL and:				
		Spiritual growth: r = 0.16		0.02		
		Interpersonal relations: r = 0.16		0.05		
		Antidepressants: r = −1.13		0.04		
		Flu vaccines: r = −0.15		0.01		
		Comparison of means (t-test)				
		HL score by:				
		Planning status of pregnancy				
		Yes: M = 76.73 (SD = 29.86)		0.01		
		No: M = 68.15 (SD = 29.77)				
		Medication use during pregnancy				
		Yes: M = 79.05 (SD = 28.20)		<0.01		
		No: M = 63.80 (SD = 31.23)				

CI = Confidence interval; DV = Dependent variable; HL = Health literacy; IV = Independent variable; M = Mean; SD = Standard deviation; r = Pearson coefficient; Rho = Spearman coefficient; OR = Odds ratio; Δ = Delta.

## Data Availability

Data sharing is not applicable to this article.

## Supplementary Materials

The following are available online at https://www.mdpi.com/article/10.3390/ijerph18073847/s1, Table S1: Ethnicity and Education and Table S2: Quality Assessment.
