# Supplementary material for: Health Literacy in Pregnant Women: A Systematic Review
Source: Int J Environ Res Public Health. 2021 Apr 6;18(7):3847. doi: 10.3390/ijerph18073847 (PMC8038834; doi:10.3390/ijerph18073847)
Supplement: Supplementary file 1 [file ijerph-18-03847-s001.zip › Supplementary Material/Supplementary S1 Education and Ethnicity.docx]

| Supplementary S1 Education and Ethnicity of study participants | | | | |
| --- | --- | --- | --- | --- |
| **1st author** | **Education** | **N (%)** | **Ethnicity** | **N (%)** |
| Delanoe et al., 2016 | ≤High school  College  University | 7 (16%)  13 (30%)  24 (54%) | Not provided |  |
| Delanoe et al., 2016a | No high school  High school diploma  Professional diploma  Collegial diploma  University degree | 4 (1.2%)  25 (7.2%)  61 (17.6%)  88 (25.4%)  168 (48.6%) | White  African or African American  Latin American  Arab  Chinese  Filipino  Korean  Other | 319 (92.2%)  4 (1.2%)  5 (1.4%)  8 (2.2%)  2 (0.6%)  1 (0.3%)  1 (0.3%)  6 (1.8%) |
| Duggan, et al., 2014 | Secondary  Tertiary | 119 (29.5%)  285‬ (70.5%) | White – Irish  White – other  Asian – Irish  Asian – other | 348 (86.1%)  53 (13.1%)  1 (0.3%)  2 (0.5) |
| Lupattelli et al., 21 | <High school  High school  >High school  Other education | 244 (4.9%)  1442 (28.8%)  2764 (55.3%)  549 (11%) | Not provided |  |
| Sahin et al., 2020 | Literate  Primary school  Middle school  High school  ≥University | 5 (1.5%)  58 (17.8%)  72 (22.1%)  89 (27.3%)  102 (31.3%) | Not provided |  |
| Sheinis et al., 2018 | ≤High school  Certificate/diploma  University Bachelor’s degress  ≥Masters | 11 (8.1%)  24 (17.8%)  51 (37.8%)  49 (36.3%) | Not provided |  |
| Sheinis et al., 2018a | ≤High school  Certificate/diploma  Bachelor’s degress  ≥Masters | 12 (5.5%)  26 (11.9%)  111 (50.9%)  69 (31.7%) | African  Middle Eastern  Asian  Caribbean  European  Latin, Central and South American  North American Aboriginal  Oceanic  Other  More than one response  No response | 4 (1.8%)  12 (5.5%)  44 (20.2%)  7 (3.2%)  125 (57.3%)  8 (3.7%)  6 (2.8%)  1 (0.5%)  2 (1%)  3 (1.4%)  6 (2.8%) |
| Shieh et al., 2009 | <12 years  =12 years  >12 years | 49 (34.3%)  61 (42.6%)  33 (23.1%) | Black  White  Hispanic  Others | 86 (60.1%)  35 (24.5%)  11 (7.7%)  11 (7.7%) |
| Shieh et al., 2010 | <12 years  =12 years  >12 years | 49 (34.3%)  61 (42.6%)  33 (23.1%) | Black  White  Hispanic  Others | 86 (60.1%)  35 (24.5%)  11 (7.7%)  11 (7.7%) |
| Van Schendel et al., 2016 | Missing  Low  Intermediate  High | 2 (0.1%)  92 (8.4%)  327 (30.0%)  670 (61.5%) | Missing  Dutch  Other western  Non-western | 14 (1.2%)  807 (74%)  129 (11.8%)  141 (13%) |
| Van Schendel et al., 2017 | Low  Intermediate  High | 42 (6.2%)  202 (29.6%)  438 (64.2%) | Missing  Dutch  Other western  Non-western | 5 (0.7%)  528 (77.4%)  75 (11%)  74 (10.9%) |
| Wilson et al., 2012 | Missing  <11th grade  11th grade  >11th grade | 1 (3%)  15 (44%)  17 (50%)  1 (3%) | Black | 34 (100%) |
| Yee et al., 2014 | <High school  High school grade/GED  Some College  ≥ College graduate | 15 (9.8%)  39 (26%)  54 (35.8%)  43 (28.4%) | Non-Hispanic white  Non-Hispanic black  Hispanic  Other | 11 (7.3%)  80 (53.3%)  42 (28%)  17 (11.4%) |
| You et al., 2012 | <8th grade  9th–11th grade  12th grade or GED  1–3 years of college or technical school  College graduate | 1 (0.9%)  12 (10.9%)  26 (23.6%)  50 (45.5%)  21 (19.0%) | African-American  Caucasian  Hispanic  Asian  Other | 65 (59%)  6 (5.5%)  33 (30%)  3 (2.7%)  3 (2.7%) |
